# Supplementary material for: Influenza A Virus Infection Reactivates Human Endogenous Retroviruses Associated with Modulation of Antiviral Immunity
Source: Viruses. 2022 Jul 21;14(7):1591. doi: 10.3390/v14071591 (PMC9320126; doi:10.3390/v14071591)
Supplement: Supplementary file 1 [file viruses-14-01591-s001.zip › MotifAnalysis.zip!/MotifOutput_SC35MdNS1/homerResults/motif16.similar.html]

motif16

## Information for motif16

C
G
T
A
A
C
T
G
C
G
T
A
A
G
T
C
A
C
G
T
A
C
G
T
A
C
G
T
A
C
T
G
A
G
T
C
A
C
G
T
A
G
T
C
A
C
G
T
  
Reverse Opposite:  

C
G
T
A
A
C
T
G
C
G
T
A
A
C
T
G
A
G
T
C
C
G
T
A
C
G
T
A
C
G
T
A
A
C
T
G
A
C
G
T
A
G
T
C
A
C
G
T
  

|  |  |
| --- | --- |
| p-value: | 1e-15 |
| log p-value: | -3.679e+01 |
| Information Content per bp: | 1.530 |
| Number of Target Sequences with motif | 5.0 |
| Percentage of Target Sequences with motif | 6.49% |
| Number of Background Sequences with motif | 0.5 |
| Percentage of Background Sequences with motif | 0.00% |
| Average Position of motif in Targets | 129.0 +/- 60.6bp |
| Average Position of motif in Background | 167.0 +/- 0.0bp |
| Strand Bias (log2 ratio + to - strand density) | 10.0 |
| Multiplicity (# of sites on avg that occur together) | 1.00 |
| Motif File: | file (matrix) reverse opposite |

### Similar de novo motifs found

|  |  |  |  |  |  |  |  |
| --- | --- | --- | --- | --- | --- | --- | --- |
| Rank | Match Score | Redundant Motif | P-value | log P-value | % of Targets | % of Background | Motif file |
| 1 | 0.827 | A C T G C T G A A G T C C G A T A C T G A C G T T A G C A G T C G A C T A G T C | 1e-10 | -23.030494 | 9.09% | 0.17% | motif file (matrix) |
| 2 | 0.694 | C T A G C G T A A G T C C G A T C A G T C G A T A G T C T A G C | 1e-8 | -18.730700 | 23.38% | 4.51% | motif file (matrix) |
